# Regulation of endosomal motility and degradation by amyotrophic lateral sclerosis 2/alsin

**DOI:** 10.1186/1756-6606-2-23

**Published:** 2009-07-24

**Authors:** Chen Lai, Chengsong Xie, Hoon Shim, Jayanth Chandran, Brian W Howell, Huaibin Cai

**Affiliations:** 1 Laboratory of Neurogenetics, National Institute on Aging, National Institutes of Health, Bethesda, MD 20892 USA; 2 Neurogenetics Branch, National Institute of Neurological Disorders and Stroke, National Institutes of Health, Bethesda, MD 20892 USA; 3 Current address: School of Medicine at Virginia Commonwealth University, Richmond, VA 23298, USA; 4 Current address: University of Edinburgh, Centre for Inflammation Research, The Queen's Medical Research Institute, 47 Little France Crescent, Edinburgh, EH16 4TJ, UK

## Abstract

Dysfunction of alsin, particularly its putative Rab5 guanine-nucleotide-exchange factor activity, has been linked to one form of juvenile onset recessive familial amyotrophic lateral sclerosis (ALS2). Multiple lines of alsin knockout (*ALS2*^-/-^) mice have been generated to model this disease. However, it remains elusive whether the Rab5-dependent endocytosis is altered in *ALS2*^-/- ^neurons. To directly examine the Rab5-mediated endosomal trafficking in *ALS2*^-/- ^neurons, we introduced green fluorescent protein (GFP)-tagged Rab5 into cultured hippocampal neurons to monitor the morphology and motility of Rab5-associated early endosomes. Here we report that Rab5-mediated endocytosis was severely altered in *ALS2*^-/-^neurons. Excessive accumulation of Rab5-positive vesicles was observed in *ALS2*^-/- ^neurons, which correlated with a significant reduction in endosomal motility and augmentation in endosomal conversion to lysosomes. Consequently, a significant increase in endosome/lysosome-dependent degradation of internalized glutamate receptors was observed in *ALS2*^-/- ^neurons. These phenotypes closely resembled the endosomal trafficking abnormalities induced by a constitutively active form of Rab5 in wild-type neurons. Therefore, our findings reveal a negatively regulatory mechanism of alsin in Rab5-mediated endosomal trafficking, suggesting that enhanced endosomal degradation in *ALS2*^-/- ^neurons may underlie the pathogenesis of motor neuron degeneration in ALS2 and related motor neuron diseases.

## Background

Amyotrophic Lateral Sclerosis (ALS) is a neurodegenerative disease caused by the selective degeneration of spinal and corticospinal motor neurons, resulting in muscle weakness and atrophy along with spastic paralysis [1,2]. One form of inherited juvenile-onset amyotrophic lateral sclerosis (ALS2) is caused by loss of function mutations in the *ALS2 *gene [3-6]. Elucidation of the function(s) of alsin is essential in [7]understanding the pathogenic mechanism of this type of motor neuron disease.

Alsin, encoded by the full-length *ALS2 *gene, contains three putative guanine-nucleotide-exchange factor (GEF) domains based on the sequence homology [4,6]. Previous studies indicate that the carboxyl-terminal vacuolar protein sorting 9 (VPS9)-like domain in conjunction with the upstream membrane occupation and recognition nexus (MORN) motifs specifically facilitates GDP/GTP exchange in Rab5 family GTPases [8,9]. Almost all reported mutations in the *ALS2 *gene result in the loss of the VPS9 domain [4,6,10-13], suggesting that the proposed Rab5 GEF activity of alsin plays a critical role in protecting motor neurons from degeneration.

Rab5 is essential in regulating organelle tethering, fusion and microtubule-dependent motility during endocytosis [14]. Early endosomes are constantly generated in the cell periphery and targeted to either the recycling or lysosome-dependent degradation pathway. The endosomes bound for the degradation route migrate to the cell center while growing in size and eventually fuse with lysosomes [15,16]. Deficiency in endosomal trafficking has been reported in mouse models of Down's syndrome and Huntington's disease [17,18]. Similarly, the dynamics of endosomal transport and fusion appear to be compromised in cells derived from *ALS2 *knockouts (*ALS2*^-/-^) mice. *ALS2*^-/- ^fibroblasts when treated with epithelium growth factor (EGF) show a delay in EGF receptor-mediated endocytosis, which is supported by a similar study on neurons treated with brain derived growth factor (BDNF) [19,20]. However, since alsin is an activator of Rac1 GTPase [9,21] and Rac1 is also involved in EGF-induced endocytosis [22], it is not clear that the delayed endocytic response in *ALS2*^-/-^cells is due to the dysfunction of Rab5 or Rac1-dependent pathway.

To directly explore the function of alsin in Rab5-dependent endosomal trafficking in neurons, we used green fluorescent protein (GFP)-tagged Rab5 as a tracer to monitor the size, motility, and degradation of endosomes in cultured neurons derived from *ALS2*^-/- ^mice [23,24]. Since Rab5-mediated endosomal trafficking is also involved in sequestration of glutamate receptors during synaptic transmission [25,26], we also investigated the role of alsin in the turnover of internalized glutamate receptors. We found that deficiency in alsin led to a significant accumulation of enlarged Rab5-associated endosomes in neurons, which was correlated with a dramatic decrease of endosomal motility and may contribute to the increased lysosome-dependent degradation of internalized glutamate receptors in *ALS2*^-/- ^neurons. Our findings reveal a novel function of alsin in negatively regulating Rab5-mediated endosomal trafficking and suggest that increased degradation of internalized cargo proteins may contribute to the pathogenesis of ALS2 and related motor neuron diseases.

## Methods

### Animals

The generation of *ALS2*^-/- ^mice was described previously [23]. The mice were housed in a 12-hour light/dark cycle and fed regular diet *ad libitum*. The experimental protocols utilized in this paper are in accordance with guidelines of the Institutional Animal Care and Use Committees of the National Institute of Child Health and Human Development.

### Expression Constructs

The full-length mouse *ALS2 *cDNA clone was obtained by a combination of 5' RACE PCR and screening of a mouse brain cDNA library (Origene, Rockville, MD). The cDNA was then subcloned into modified pRK5 expression vectors [27], which were tagged at the N-terminus with DsRed (mono), a red fluorescent protein (RFP), or EGFP obtained from Invitrogen (Grand Island, NY).

### Neuron Culture and Transfection

Primary hippocampal cultured neurons were derived from postnatal day 0 pups as described previously [23]. One million dissociated neurons were plated onto each well of 6-well plates (Becton Dickinson Labware, Bedford, MA) or Delta T dishes (Bioptechs Inc. Butler, PA) pre-coated with poly-D-lysine (PDL, Sigma, St Louis, MO). For immunofluorescence analysis, 0.3 million dissociated neurons were plated on cover slips (Glaswarenfabrik Karl Hecht KG, Sondhein, Germany) pre-coated with PDL. Cultures were maintained in Basal Medium Eagle (Sigma) supplemented with B27, N2, penicillin/streptomycin and L-glutamine (Invitrogen), and incubated in a humidified environment at 37°C and 5% CO_2_. 70% of the media was changed every 3 d. Hippocampal neurons were transfected 10 d after culture (DIV10) using Effectene transfection reagent (Qiagen, Germany) according to manufacturer instructions. Neurons were fixed with 4% paraformaldehyde for 10–15 min 4 d after transfection and stored at 4°C for immunocytochemical analysis.

### Time-lapse Fluorescence Microscopy and Image Capture

Dissociated hippocampal neurons were cultured on PDL and ECL-coated Delta T dishes and perfused with fresh culture medium supplemented with 20 mM HEPES (pH 7.3) during time-lapse imaging. Fluorescence microscopy and digital image acquisition were carried out using a Nikon ECLIPSE TE200 inverted fluorescence microscope equipped with a Nikon 60 × 1.2 numerical aperture water immersion objective and a cooled CCD camera driven by the DeltaVision Real-Time Restoration Imaging System (Applied Precision, LLC). The GFP and RFP-tagged proteins were excited at 488 nm and 535 nm, and emitted fluorescence was collected through a 525–30 and a 610–75 nm band pass emission filter, respectively. To minimize cell phototoxicity, we used a computer-driven automatic shutter to achieve minimum illumination. An image series constituted 150 time points at 1 sec intervals, and each recording lasted 5 min. The kymography of vesicle movement was prepared using ImageJ (NIH, Bethesda, MD). For some experiments, post time-lapse recording, the cells were fixed and processed for immunostaining with an anti-MAP2 antibody (Sigma).

### Analysis of Endosomal Transport

The movement of Rab5-positive particles was analyzed with the SoftWoRx Image Analysis System software (Applied Precision, LLC). The particle diameters were measured using the Standard Two Point method, and the total movement distance of one particle (diameter > 0.1 μm) for 150 frames was measured with the leapfrog method. The total distance (μm) that a particle traveled was divided by the duration of the movement (second) to obtain the average speed. At least three independent fields were assessed for every experiment. Data was collected from at least three individual experiments.

### Immunocytochemistry

Fixed hippocampal neurons were permeabilized with 0.1% Triton X-100 in PBS for 10 min, blocked with 10% normal goat serum at room temperature for 1 hour, and incubated with primary antibody at 4°C overnight. The next day, cells were washed three times with PBS, incubated with Alexa 488 or Alexa 568 conjugated secondary antibody (Invitrogen) at room temperature for one hour, and mounted with Pro-Long Gold anti-fade regent (Invitrogen) after washing three times with PBS.

### Confocal Imaging

A Zeiss LSM510 META Axioplan2 Confocal Microscope (Carl Zeiss, Germany) was used with constant settings of laser power, detector gain, amplification gain, pinhole, and offset. Images were scanned using either a 63 × 1.4 NA oil objective or 100 × 1.4 NA oil objective lens. A zoom factor of 2 was used to obtain maximum resolution in 100 × images. Some images were acquired in z-series stack scans at 1 μm intervals from individual fields to determine the protein-protein co-localization between Rab5 and LAMP1.

### Co-localization Analysis

As described previously [28], confocal images processed for co-localization analysis were collected using a 100 × 1.4 NA oil objective lens; zoom setting, 2.0; z-step, 1 μm; and image size, 1042 × 1042 pixels. Excitation filters were used at 488 nm and 535 nm, and emitted fluorescence was collected through a 525–30 and a 610–75 nm band pass emission filter, respectively. During image collection, the gain and offset level were set so that the full range of pixel intensities was used (0–255) with very little saturation at either end of the intensity range. The numbers and total area covered by co-localized pixels were obtained from co-localized table efferent from each image.

### Cell Surface Protein Internalization Assay

As described previously [29], cortical neurons after 21d in culture were used for cell surface protein internalization assay. Briefly, cells were labeled with EZ-Link Sulfo-NHS-SS-Biotin (Pierce, Rockford, IL) for 2 min at 37°C. After washing in Tris-buffered saline, neurons were incubated in medium with or without 100 μM AMPA for 15 min. Membrane protein trafficking was halted by rapid cooling to 4°C. Biotinylated proteins remaining on the cell surface were stripped of biotin by the non-permeant reducing agent glutathione (150 mM glutathione, 150 mM NaCl, pH 8.75). Glutathione was subsequently neutralized by 50 mM iodoacetamide in PBS with magnesium and calcium. Cells were lysed in modified RIPA buffer (1% Triton X-100, 0.5% SDS, 0.5% deoxycholic acid, 50 mM NaPO4, 150 mM NaCl, 2 mM EDTA, 50 mM NaF, 10 mM sodium pyrophosphate, 1 mM sodium orthovanadate, and protein inhibitor cocktail). To examine the effect of endosomal degradation in internalized proteins, neuronal cultures were pretreated with 100 μg/ml leupeptin (Sigma) 1 hr prior to biotinylation and kept incubated with leupeptin through the rest of the experiment. The homogenates were centrifuged at 14, 000 × *g *for 15 min at 4°C. 10% of the supernatant was saved as total protein input and the remaining supernatant was incubated with 100 μl of 50% UltraLink Immobilized NeutrAvidin agarose beads (Pierce) for 3 hr at 4°C. The beads were washed five times with RIPA buffer, and then bound proteins were eluted with SDS sample buffer by boiling for 10 min. Total protein and isolated biotinylated proteins were analyzed by immunoblotting with anti-GluR2, GluR2/3, GluR1, NR1, ALS2, and β-tubulin antibodies. Protein bands were visualized by ECL (Pierce) and quantified by the Scion Image System (Scion, Frederick, MD).

### Data Analysis

Because it is not certain that data are normally distributed, the non-parametric Mann-Whitney test (SAS Institute Inc., Cary, NC) was used to compare the two groups and a *p *value of 0.05 was used as the criterion for significance.

## Results

### Accumulation of enlarged Rab5-associated endosomes in *ALS2*^-/- ^hippocampal neurons

**Figure 1:**
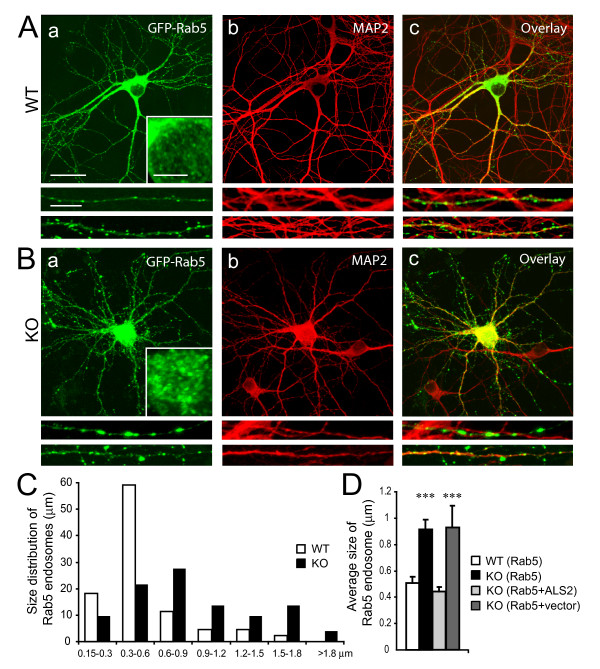
**Increased accumulation of enlarged Rab5-associated endosomes in *ALS2*^-/- ^hippocampal neurons**. A-B. Representative images of wild-type (WT; *A*) and *ALS2*^-/- ^(KO; *B*) hippocampal neurons transfected with GFP-Rab5 (a, green) and counterstained with an antibody against MAP2 (b, red). Fragments of soma (inset), axon (middle panel) and dendrite (bottom panel) were enlarged to reveal the distribution and morphology of Rab5-positive vesicles. Panel 'c' is an overlay. Scale bar: 20 μm (main) and 5 μm (inset, middle and bottom panels). C. Histogram reveals the distribution of Rab5-associated vesicles within each size category in WT and KO neurons (n = 5). D. Histogram reveals the average size of Rab5-associated vesicles (n > 45) in wild-type (WT (Rab5)), *ALS2*^-/- ^(KO (Rab5)), ALS2-transfected *ALS2*^-/- ^(KO (Rab5+ALS2)) and DsRed-transfecetd *ALS2*^-/- ^(KO (Rab5+vector)) neurons. Error bars represent SEM. *** *P *< 0.001.

To investigate whether alsin-deficiency affects the morphology of early endosomes, we expressed GFP-Rab5 in hippocampal neurons derived from wild-type and *ALS2*^-/- ^mice. In wild-type neurons, a ubiquitous distribution of GFP-Rab5-associated vesicles was observed in soma (Figure 1Aa, inset), axons (Figure 1Aa and 1Ac, middle panel), and dendrites (Figure 1Aa and 1Ac, bottom panel), with approximately 80% of GFP-Rab5-positive vesicles smaller than 0.6 μm in diameter (average diameter = 0.5 ± 0.05 μm, Figure 1C–D) [24]. In contrast, significantly enlarged GFP-Rab5-positive vesicles (average diameter = 0.9 ± 0.07 μm, *p *< 0.001, Fig. 1C and 1D) were observed in soma (Figure 1Ba, inset), axons (Figure 1Ba and 1Bc, middle panel), and dendrites (Figure 1Ba and 1Bc, bottom panel) of *ALS2*^-/- ^neurons. Except for the morphological changes of Rab5-positive vesicles, *ALS2*^-/- ^neurons appeared healthy and maintained normal structures of nuclei, dendrites and axons (Figure 1Bb). Furthermore, reintroduction of full-length ALS2 or VPS9 domain expression construct, but not a control vector, into *ALS2*^-/- ^neurons significantly reduced the number of enlarged GFP-Rab5-positive vesicles to a level comparable to wild-type neurons (Figure 1D).

**Figure 2:**
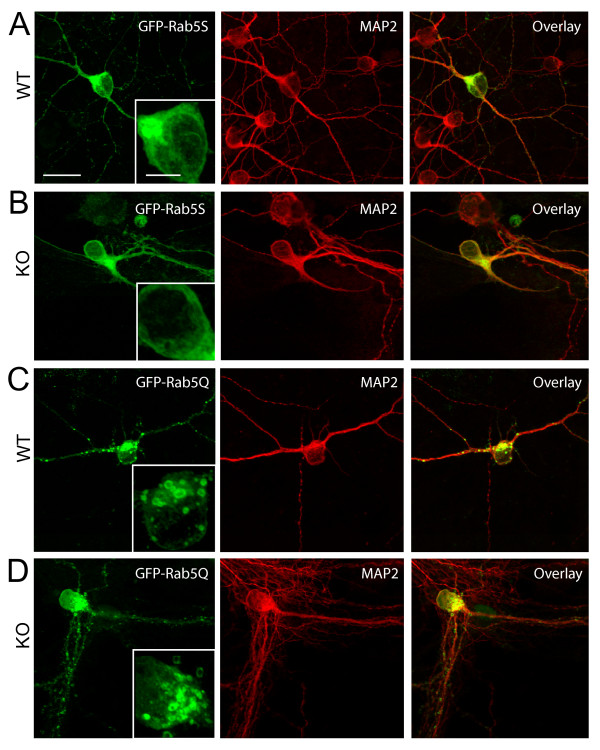
**The Enlargement of Rab5-positive endosomes in *ALS2*^-/- ^neurons may correlate with increased Rab5 activities**. Representative images of wild-type (WT) and *ALS2*^-/- ^(KO) hippocampal neurons transfected with GFP-Rab5S (A and B) or GFP-Rab5Q (C and D). Fragments of soma (inset) were enlarged to reveal the distribution and morphology of Rab5-positive vesicles. Scale bar: 20 μm (main) and 5 μm (inset).

To test whether the increased accumulation of enlarged Rab5-associated endosomes in *ALS2*^-/- ^neurons was dependent on Rab5 activities, we transfected GFP-Rab5A-S34N (GFP-Rab5S), a dominant-negative mutant of Rab5 [30], into wild-type and *ALS2*^-/- ^neurons (Figure 2A–B). The size of GFP-labeled endosomes in *ALS2*^-/-^neurons (Figure 2B) was similar to wild-type neurons (Figure 2A) after introduction of GFP-Rab5S, suggesting that the enlargement of Rab5-associated endosomes in *ALS2*^-/- ^neurons is resulted from Rab5-mediated endosome fusion. As a control, over-expression of a constitutive active form of Rab5, Rab5A-Q79L (Rab5Q), led to the enlargement of Rab5-positive endosomes in both wild-type and *ALS2*^-/- ^neurons (Figure 2C–D).

### Decreased motility of Rab5-positive endosomes in *ALS2*^-/- ^hippocampal neurons

**Figure 3:**
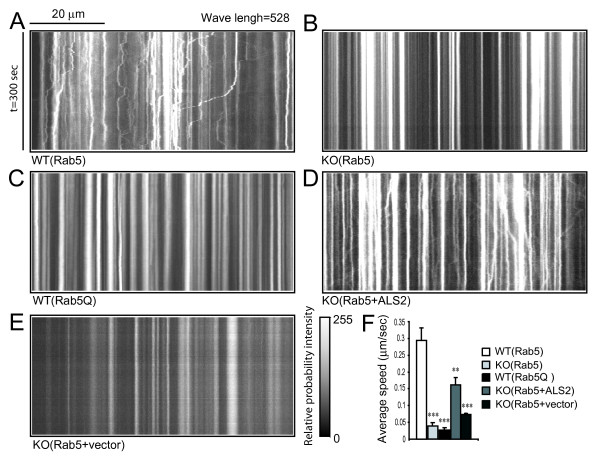
**Decreased motility of Rab5-associated endosomes in *ALS2*^-/- ^neurons**. *A-E*. Representative kymographs of GFP-Rab5-positive endosome movement in wild-type (WT (Rab5), A), *ALS2*^-/- ^(KO (Rab5), B), Rab5Q-transfected wild-type (WT (Rab5Q), C), DsRed-ALS2 transfected *ALS2*^-/- ^(KO (Rab5+ALS2), D), and DsRed-vector alone transfecetd *ALS2*^-/- ^(KO (Rab5+vector), E) hippocampal neurons. Each kymograph summarizes the spatial positions (x-axis) of Rab5 endosomes at certain time points (y-axis) in one video field. *F*. Histogram shows the average speed of the Rab5-positive endosomes (*n *> 30) in WT (Rab5), KO (Rab5), WT (Rab5Q), KO (Rab5+ALS2), and KO (Rab5+vector) neurons. Error bars indicate SEM. **P < 0.05. ***P < 0.01.

Rab5 is also involved in microtubule-based endosomal transport [31]. To examine whether the transport of Rab5-positive endosomes is impaired in *ALS2*^-/- ^hippocampal neurons, we measured the motility of Rab5-positive endosomes by time-lapse fluorescence imaging in neurons transfected with GFP-Rab5 (Figure 3 and see Additional files). As shown in the kymographs that summarized the spatial position (x-axis) of each Rab5 vesicle over time (y-axis), Rab5-positive endosomes moved in a rapid (average speed = 0.29 ± 0.04 μm/sec), frequent, and asynchronous manner in either anterograde or retrograde direction in dendrites of wild-type neurons (Figure 3A, F and see Additional file 1). In contrast, the movement of GFP-Rab5-positive vesicles in *ALS2*^-/-^neurons was significantly slower (Figure 3B and 3F, average speed = 0.04 ± 0.005 μm/sec, *p *< 0.001) than that of wild-type controls. In fact, most of the large Rab5-positive endosomes in *ALS2*^-/- ^neurons (diameter > 1 μm) were stationary (Figure 3B and see Additional file 2). A similar reduced motility of Rab5-positive endosomes was also observed in wild-type neurons transfected with Rab5Q (Figure 3C, F and see Additional file 3), indicating that the slower movement of endosomes in *ALS2*^-/- ^neurons may be caused by an increased endosomal fusion. Reintroduction of *ALS2 *significantly increased the motility of Rab5-positive vesicles in *ALS2*^-/- ^neurons (Figure 3D, F and see Additional file 4), while co-transfection of a control vector (DsRed-vector) with GFP-Rab5 had no such effect (Figure 3E, F and see Additional file 5). Taken together, our data demonstrate that loss of alsin severely disrupts Rab5-dependent endosomal trafficking in neurons.

### Increased co-localization of Rab5 and LAMP1 in large Rab5-associated endosomes of *ALS2*^-/- ^hippocampal neurons

**Figure 4:**
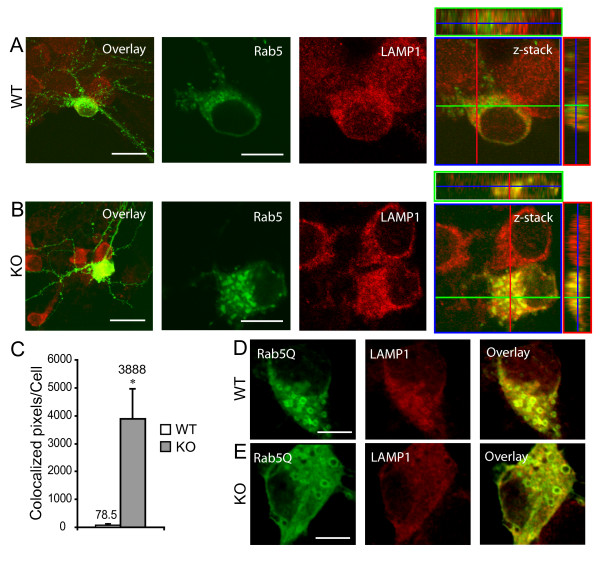
**Increased co-localization of LAMP1 with Rab5-associated vesicles in *ALS2*^-/- ^neurons**. (A-D) A lower (left) or higher magnification of Rab5-transfected wild-type (WT, A), or *ALS2*^-/- ^(KO, B) neurons co-stained with LAMP1 (red) and GFP (green) antibodies. Co-localization of LAMP1 (red) and Rab5 (green) in WT and KO neurons was revealed by z-series stack images. Scale bars = 20 μm (left) and 10 μm (the rest) respectively. C. Quantitative analysis of co-localization of LAMP1 and Rab5 immuno-reactivity in WT (*n *= 4) and KO (*n *= 7) neurons. Error bars indicate SEM. * *P *< 0.05. D-E. In the soma, LAMP1 (red) and Rab5Q (green) were co-localized in either GFP-Rab5Q-transfected wild-type (WT, D) or *ALS2*^-/- ^(KO, E) neurons. Scale bar = 20 μm.

Many large Rab5-positive endosomes resulting from fusion of early endosomes are subsequently targeted to lysosomes for degradation and constitutive activation of Rab5 enhances the conversion from endosomes to lysosomes [32]. To examine whether the large Rab5-associated endosomes in *ALS2*^-/- ^hippocampal neurons are more likely to be targeted to the late endosome and lysosome-dependent degradation pathway, we stained GFP-Rab5 transfected *ALS2*^-/- ^neurons with an antibody against lysosome membrane protein 1 (LAMP1), a marker for late endosomes/lysosomes [33,34]. We found a substantial increase of co-localization of Rab5 and LAMP1 immunofluorescence signals in the soma of *ALS2*^-/-^neurons (Figure 4B–C), which reflects an enhanced conversion of early endosomes to lysosomes for endosomal degradation. In contrast, only very sparse co-localization of Rab5 and LAMP1 staining was observed in wild-type neurons (Figure 4A and 4C, *p *< 0.05). Moreover, transfection of GFP-Rab5Q induced a similar increase of co-localization of LAMP1 and Rab5 in the soma of both wild-type and *ALS2*^-/- ^neurons (Figure 4D–E), indicating that increased Rab5-mediated fusion of endosomes promotes the degradation of early endosomes.

### Increased Degradation of Internalized Glutamate Receptors in AMPA-Treated *ALS2*^-/- ^Neurons

**Figure 5:**
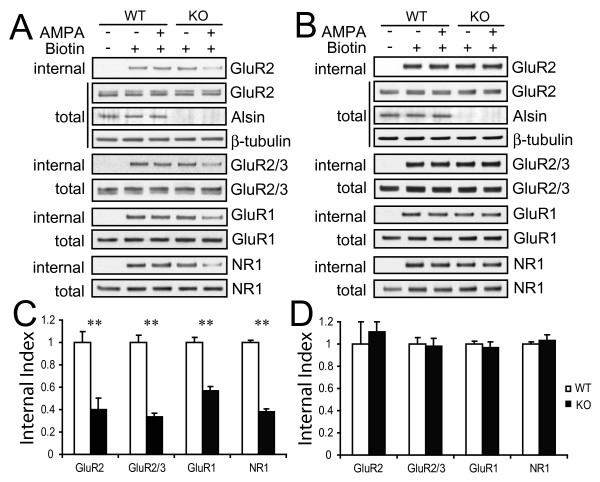
**Increased degradation of glutamate receptors in *ALS2*^-/- ^neurons following AMPA treatment**. A. The biotinylated internalized cell surface proteins were affinity purified and immunoblotted with antibodies against glutamate receptors: GluR1, GluR2, GluR2/3, and NR1 (labeled as "internalized"). The neurons were treated with mock (AMPA-) or AMPA (AMPA+) prior to the biotinylation. The expression of each glutamate receptor and β-tubulin in total cell lysate (labeled as "total") was also blotted to normalize the accumulation of internalized proteins. B. Similar to A, except that neurons were pre-incubated with lysosome inhibitor leupeptin. C. Histogram reveals a significant decrease of internalized glutamate receptors in KO neurons as compared with WT controls (*n *> 6). Error bars represent SEM. ** *P *< 0.01. D. Histogram shows that in the presence of leupeptin, no significant alteration of internalized glutamate receptors was observed between WT and KO neurons (*n *> 6). Error bars represent SEM.

To investigate whether endocytosis of cell surface receptors was altered in *ALS2*^-/- ^neurons, we treated cultured cortical neurons with AMPA, an analog of glutamate to induce the internalization of glutamate receptors that depends on the Rab5-mediated endosomal trafficking pathway [25,26]. We first examined the AMPA-induced internalization of glutamate receptors in *ALS2*^-/- ^neurons. Internalized proteins were labeled with biotin, which were then affinity-purified and immunoblotted with antibodies against glutamate receptors (Figure 5A–B). A significant reduction of internalized receptors was observed in AMPA-treated *ALS2*^-/- ^neurons compared with AMPA-treated wild-type controls (Figure 5A and 5C, *p *< 0.01). In contrast, no significant alterations in internalized glutamate receptor proteins were observed between mock-treated wild-type and *ALS2*^-/- ^neurons (Figure 5A). This reduction of internalized glutamate receptors in *ALS2*^-/- ^neurons may result from either decreased endocytosis of cell surface receptors or increased degradation of internalized proteins. To differentiate these two possibilities, prior to AMPA application we treated neurons with leupeptin, a specific lysosome inhibitor, to block lysosome-mediated degradation of internalized glutamate receptors [35]. We found no significant difference in internalized glutamate receptors between wild-type and *ALS2*^-/- ^neurons after treatment with both leupeptin and AMPA (Figure 5B and 5D). Together, these data suggest that the endocytosis of cell surface receptors might not be affected in *ALS2*^-/- ^neurons, while the lysosome-mediated degradation of glutamate receptors is significantly enhanced in these neurons.

## Discussion

Autosomal recessive mutations in the *ALS2 *gene lead to a clinical spectrum of motor dysfunction including juvenile onset amyotrophic lateral sclerosis (ALS2), primary lateral sclerosis and hereditary spastic paraplegia [4,6,11]. Multiple *in vitro *biochemical and cell biology assays suggest that alsin dysfunction affects endosomal trafficking through Rab5-mediated mechanism [8,9,19,20]. In this report we extended those early studies and revealed some novel functions of alsin in negatively regulating endosomal trafficking and degradation. We found that alsin-deficiency led to increased endosomal fusion and degradation, and decreased endosomal motility. Concomitantly, we demonstrate that the degradation of internalized AMPA receptors was significantly enhanced in *ALS2*^-/- ^neurons. Taken together, our data indicate that excessive endosomal degradation in *ALS2*^-/- ^neurons may contribute to the pathogenesis of ALS2 and related motor neuron diseases.

How alsin affects the Rab5-mediated endocytosis is a focal point in understanding the function of alsin in motor neuron degeneration. To determine whether alsin activates Rab5 and promotes endosomal fusion, multiple groups have transfected full-length alsin or truncated forms containing either the VPS9 domain alone, or the VPS9 domain with other domains. While all forms can stimulate the release of bound GDP from Rab5 subfamily GTPases in a cell free GDP dissociation assay, over-expression of full-length alsin is unable to stimulate Rab5-mediated endosomal fusion as efficiently as truncated forms lacking the chromosome condensation 1 (RCC1) like domains (RLD) [8,9]. Analyses of a range of alsin deletion constructs suggest that multiple domains influence its Rab5-GEF activity [8]. While much of the current literature suggests that the DH/PH and VPS9 domains with MORN motifs of alsin are necessary to promote Rab5 activity in the endocytic pathway, a regulatory role for the RLD domain has been proposed [8]. Recently, two motor neuron disease-related missense mutations (C156Y and G540E) have been identified within the RLD domain, indicating that this domain also plays an important role in the normal function of alsin [36,37]. The RLD domain consists of a seven-bladed propeller formed from internal amino acid repeats [38]. When only the RLD domain of alsin is expressed in cell lines, it displays a cytosolic distribution similar to that of over-expression of full-length alsin [8,9]. By contrast, over-expression of alsin lacking the RLD domain results in endosomal localization [8,39]. Therefore, the RLD domain of alsin may prevent the association of alsin with early endosomes and act as a negative regulator of Rab5 mediated endosomal fusion. In line with this notion, loss of alsin may expose more Rab5 to the upstream activators and downstream effectors, and enhance Rab5-mediated endosomal fusion, resulting in an increased accumulation of enlarged endosomes as observed in alsin-deficient neurons. Consistently, a recent report revealed that the accumulation of enlarged insulin-like growth factor 1 (IGF1) receptor-containing early endosomes following IGF1 treatment is 1000 times higher in *ALS2*^-/- ^neurons compared to wild-type controls [19]. The same report, however, also showed that the cytosol prepared from *ALS2*^-/- ^mouse brains dramatically reduced its ability to promote Rab5-dependent early endosomal fusion in a cell free assay, which is apparently in conflict with its cell-based assays [19]. The different observation in the cell-free assay [19] may reflect a depletion of Rab5 downstream effectors in the cytosol of *ALS2*^-/- ^neurons due to an elevation of Rab5 activity.

Since we only disrupted the 2^nd ^coding exon of *ALS2 *gene in our knockout mice, an alternative explanation for the observed abnormal aggregation of Rab5-positive vesicles in *ALS2*^-/- ^neurons is due to the presence of more active N-terminal truncated form of alsin lacking the RLD domain. However, this hypothesis is unlikely to be true, since no short forms of alsin was detected in *ALS2*^-/- ^mouse brain lysates using an antibody specifically against the C-terminal of alsin [23]. In addition, a recent study indicates that ALS2CL, an alsin homologous protein [40], selectively interacts with the VPS9 domain of alsin and prohibits the enlargement of early endosoms induced by the N-terminal truncated alsin [41]. Therefore, even if there is a trace of N-terminal truncated alsin left in the *ALS2*^-/- ^neuron, it may not be capable to facilitate Rab5-depedent endsomal fusion in the presence of ALS2CL.

Activation of Rab5 is also involved in endosomal transport along the microtubule lattice [31]. Two previous reports show that the internalization of epithelium growth factor (EGF) and brain derived neuron growth factor (BDNF) receptors was slowed down in alsin-deficient fibroblasts and neurons [19,20]. To further investigate the role of alsin in endosomal transport, we traced the movement of Rab5-positive vesicles by time lapse imaging in *ALS2*^-/- ^neurons. We found that the motility of Rab5-associated endosomes was significantly decreased in *ALS2*^-/- ^neurons. Since disruption of microtubule-based endosomal transport causes the enlargement of Rab5-associated endosomes [31], it is possible that the accumulation of enlarged Rab5-positive vesicles observed in *ALS2*^-/- ^neurons is due to the dysfunction of microtubule-based vesicle transport. However, since a similar reduction of endosomal motility was also observed in Rab5Q-transfected wild-type neurons, our data argue that the increased endosomal fusion may play a main role in causing the endosomal motility defects in *ALS2*^-/- ^neurons. It is of interest to determine the interplay between the endosomal fusion and transport. It also remains to determine how alsin regulates Rab5-mediated endosomal transport. Rab5 regulates both attachment and transport of early endosomes along microtubules in a process dependent on the activity of phosphatidylinositol-3-OH kinase, hVPS34 [31]. Since multiple domains of alsin, including the MORN motifs, are predicted to bind phospholipids based on sequence homology [4,6], it is possible that alsin acts as a scaffold protein to assemble the protein complex, which is important in endosomal transport.

Early endosomes targeted for the lysosome-dependent degradation pathway keep growing in size while migrating to the cell center [16]. Consistently, we found extensive co-localization of lysosome marker LAMP1 with Rab5-positive vesicles in the soma of *ALS2*^-/- ^neurons, suggesting an increase of endosomal degradation. Since the internalization and degradation of glutamate receptors are both mediated by the Rab5-dependent endocytic pathway [25,26], we quantified the internalization and degradation of glutamate receptors *ALS2*^-/-^neurons. We found an increased degradation of internalized glutamate receptors in *ALS2*^-/- ^neurons. Many studies have suggested that down-regulation of calcium-impermeable GluR2-containing AMPA type glutamate receptor complexes contributes to motor neuron degeneration [42]. We have reported previously that the cell/synaptic surface presentation of GluR2 is selectively decreased in *ALS2*^-/- ^neurons following AMPA treatment, which renders *ALS2*^-/- ^neurons more vulnerable to glutamate receptor-mediated toxic stress [43]. The selective down-regulation of GluR2 at cell/synaptic surface of *ALS2*^-/- ^neurons may likely result from the increased degradation of internalized GluR2 and the deficiency in targeting the intracellular pool of GluR2 to the plasma membrane.

Endosome dysfunction has emerged as a common pathogenic pathway of many neurodegenerative diseases [44]. Neurons rely heavily on endocytosis to communicate with each other or peripheral tissues for signal transduction and survival. The highly polarized structure of neurons separates events during endocytosis at greater distance, which may render neurons more vulnerable to disturbance in the endocytic pathway. The transport of endosomes and other organelles and their cargos into the dendrites are essential to the pattern of dendritic branching and normal function of neurons [7,45]. Here, the decreased motility of endosomes in *ALS2*^-/- ^neurons may very likely affect the transport of signaling endosomes that carry signals for survival and differentiation of motor neurons. The increased degradation of internalized cargo proteins, including neurotransmitter and neurotrophic receptors may affect the synaptic transmission and intracellular signaling transduction in *ALS2*^-/- ^neurons, which may underlie the pathogenic mechanism of motor neuron degeneration in ALS2 and related motor neuron diseases.

## Abbreviations

ALS2: amyotrophic lateral sclerosis 2; KO: knockout; GEF: guanine-nucleotide-exchange factor; VPS9: vacuolar protein sorting 9; LAMP1: lysosome membrane protein 1; GluR: glutamate receptors; NR: N-Methyl-D-aspartate receptor.

## Competing interests

The authors declare that they have no competing interests.

## Authors' contributions

CL: conception and design, collection of data, data analysis, manuscript writing, discussion. CX: neuronal culture and transfection. HS: molecular genetics, construct amplification. JC: discussion. BWH: design, discussion. HC: conception and design, financial support, data analysis, manuscript writing. All authors read and approved the final manuscript.

## Supplementary Material

Additional file 1 **Trafficking of Rab5-associated endosomes in wild-type neurons**. Representative image sequence of Rab5-positive endosomes in wild-type hippocampal neurons transfected with GFP-Rab5. All movies in this paper cover an observation interval of 5 min and play at 30 frames/s. The cell body was in the left of field. The scale bar indicated 10 μm. Click here for file

Additional file 2 **Compromised trafficking of Rab5-associated endosomes in *ALS2*^-/- ^neurons**. Representative image sequence of Rab5-associated endosomes in *ALS2*^-/- ^neurons transfected with GFP-Rab5. Click here for file

Additional file 3 **Endosome trafficking in GFP-Rab5Q transfected wild-type neurons**. Representative image sequence of Rab5Q-positive endosomes in wild-type neurons transfected with GFP-Rab5Q. Click here for file

Additional file 4 **Rescue trafficking of Rab5-associated endosomes in *ALS2*^-/- ^neurons**. Representative image sequence of rescued Rab5-positive endosomes in *ALS2*^-/- ^neurons co-transfected with GFP-Rab5 and DsRed-ALS2. Click here for file

Additional file 5 **Unrescued trafficking of Rab5-associated endosomes in *ALS2*^-/- ^neurons without ALS2 reintroduction**. Representative image sequence of Rab5-positive endosomes in *ALS2*^-/- ^neurons co-transfected with GFP-Rab5 and DsRed-vector. Click here for file
